# CircGRHL2 suppresses EMT and enhances sunitinib sensitivity in ccRCC via the miR-330-5p/FBXO21 axis

**DOI:** 10.1007/s00018-026-06102-7

**Published:** 2026-04-29

**Authors:** Jie Gao, Huibao Yao, Qiancheng Mao, Chu Liu, Yuanshan Cui, Junjie Zhao, Jian Ma, Jitao Wu

**Affiliations:** Department of Urology, The Affiliated Yantai Yuhuangding Hospital of Qingdao University, NO. 20 East Yuhuangding Road, Yantai, Shandong 264000 China

**Keywords:** circGRHL2, EMT, Sunitinib sensitivity, ccRCC

## Abstract

Clear cell renal cell carcinoma (ccRCC) is one of the most common cancers in the urinary system. Studies have shown that circRNAs have potential effects in a variety of human tumors. Studying the important signaling pathways and therapeutic targets of circRNAs is essential to increase understanding of ccRCC. In this present study, we clarified that hsa_circGRHL2 was dramatically decreased in ccRCC tissues and cells, which was closely related to the clinicopathological features of ccRCC patients. Functionally, circGRHL2 significantly inhibited the proliferation, migration, and invasion of ccRCC cells in vitro. Mechanistically, circGRHL2 sponges miR-330-5p, could regulate miR-330-5p to affect proliferation, migration, invasion of ccRCC cells and have effect on epithelial-mesenchymal transition (EMT). Moreover, FBXO21, could be a direct target of miR-330-5p, which further reducing P85 phosphorylation, inhibiting PI3K/AKT pathway activation, and weakening the EMT. In addition, circGRHL2 overexpression enhances the sensitivity of 769-P cells to sunitinib and enhances the ability of sunitinib treatment effects in vivo. In summary, we proposed a novel signaling network, in which circGRHL2 inhibited the progression of ccRCC via the miR-330-5p/FBXO21 axis and decreased PI3K/AKT activation.

**Supplementary Information:**

The online version contains supplementary material available at 10.1007/s00018-026-06102-7.

## Introduction

Renal carcinoma is a common urinary tract tumor, accounting for approximately 3% of all tumors [1]. Among them, clear cell renal carcinoma (ccRCC) is the most common pathological type, accounting for about 80% of renal malignant tumors [2]. Around 30% of patients with newly diagnosed ccRCC have distant metastasis with a poor prognosis. Despite the use of targeted drugs and immunotherapy, the five-year survival rate remains below 10% [3]. Moreover, the recurrence-free survival after sunitinib treatment is only about 12 months [4]. A deeper understanding of the molecular mechanisms underlying ccRCC progression is crucial for finding new therapeutic targets and improving the overall prognosis of patients.

Circular RNA (circRNA) is a type of non-coding RNA formed by reverse splicing. The application of high-throughput sequencing and bioinformatics has revealed that circRNA plays a variety of biological roles in cancer progression, including miRNA sponge, transcriptional regulation, RNA binding protein, and translational polypeptide [5]. It involves in promoting or suppressing in the progression of breast cancer, prostate cancer, and colorectal cancer [6]. In ccRCC, circPPAP2B promotes tumor progression and metastasis through the miR-182-5p/CYP1B1 axis [7]; hsa_circ_0057105 regulates the expression of COL1A1 and VDAC2 through its sponge effect on miR-577, thereby regulates the balance between epithelial-mesenchymal transition (EMT) and ferroptosis in renal cell carcinoma [8]; circSNX6 inhibits the expression of glycerophosphocholine phosphodiesterase 1 (GPCPD1) through miR-1184, leading to decreased intracellular lysophosphatidic acid (LPA) levels and promotes sunitinib resistance [9]. Despite these findings, the functional effects and regulatory mechanisms of circRNAs on ccRCC progression remain largely unknown.

EMT is a reversible cellular process that has important effects on wound healing, tumor progression, and the development of drug resistance [10]. EMT plays an indispensable role in the pathogenesis and progression of various cancers, including colorectal cancer, breast cancer, and lung cancer [11–13]. In addition, the circRNA/EMT axis has been shown to contribute to tumor progression and drug sensitivity in brain cancer, gastrointestinal cancers, gynecological cancers, and urological cancers [14]. However, there are few studies on how circRNAs regulate EMT and affect drug sensitivity in ccRCC.

In this study, we discovered a novel circRNA, circ-Grainyhead-like 2 (circGRHL2), which was significantly downregulated in ccRCC cells and tumor tissues. Functional studies showed that circGRHL2 inhibited tumor proliferation both in vitro and in vivo. Mechanistically, circGRHL2 has a miRNA sponge function, which upregulates the expression of FBXO21 by directly binding to miR-330-5p, leading to the degradation of the functional subunit P85 of PI3K through its ubiquitination function, which then inhibits the activation of PI3K/AKT pathway. The inactivated PI3K/AKT pathway attenuates EMT, therefore inhibits tumor progression. At the same time, we found that overexpression of circGRHL2 in 769-P cells reduced the IC50 of sunitinib and enhanced the drug antitumor efficacy in vivo. In summary, our data suggested that the newly discovered circGRHL2 could inhibit the proliferation of ccRCC and its sensitivity to sunitinib by targeting the miR-330-5p/FBXO21 axis.

## Method and materials

### Patients tissue samples

Paired primary ccRCC tumor tissues and adjacent non-tumor tissues were collected from 100 patients who underwent partial nephrectomy or radical nephrectomy at Yantai Yuhuangding Hospital affiliated to Qingdao University from 2020 to 2022. The distance between the non-tumor tissues and the tumor edge is greater than 2 cm. All tissues were snapped frozen in liquid nitrogen and stored in the freezer at −80℃ until use. All patients in this study did not receive radiotherapy, chemotherapy or neoadjuvant therapy before surgery, and the tissues selected were confirmed to be the clear cell renal cell carcinoma by pathological examination. All patients have signed informed consent forms, and the project has been approved by the Ethics Committee of Yantai Yuhuangding Hospital affiliated to Qingdao University. The clinical and pathological characteristics of patients are shown in Supplemental Table 1 in detail.

### Cell lines and cell culture

Human embryonic kidney cell line (HEK293T), human renal tubular epithelial cell line (HK-2) and human ccRCC cell lines (786-O, 769-P, ACHN, Caki-1, A498) were purchased from the cell bank of the Chinese Academy of Sciences. HK-2 cells were cultured in DMEM medium (BI, Israel). The 786-O, 769-P, ACHN, Caki-1, A498 and HEK293T cells were cultured in RPMI-1640 medium (BI, Israel). All the medium was supplemented with 10% fetal bovine serum (BI, Israel) and 1% penicillin/streptomycin (BI, Israel). All the cells were incubated in a cell culture incubator at 37℃ with 5% carbon dioxide.

### RNase R treatment and RNA stability assays

A total of 3 μg RNA were treated with 5 U/μg RNase R (Novoprotein, Shanghai, China) and incubated at 37 °C for 15 min. Total RNA was then extracted and analyzed by qRT-PCR. An untreated control group was included for comparison.

### Cell transfection

The main transfection methods used in this study were lentivirus transfection and siRNA transfection. Cells were infected with lentivirus at a multiplicity of infection (MOI) of 30 and screened by puromycin. The lentivirus and transfection reagent were then added to cells in logarithmic growth phase. Cells were subsequently selected with RPMI-1640 complete medium containing 2 μg/ml of puromycin for further study. For siRNA transfection, the siR-circGRHL2 and its corresponding negative control siR-NC were synthesized by General Biol Company (China). Lipofectamine 3000 (Thermo Fisher Scientific, USA) were used to transfect the ccRCC cell lines according to the manufacturer's instructions. After 6–8 h, the original culture medium was replaced with complete medium, and the transfected cells were cultured for an additional 24–48 h for further study. Oligonucleotide sequences: si-circRNA-GCCACAGAGAAACUGCCUUUU-AAGGCAGUUUCUCUGUGGCUU-General Biol Company; miR-330-5p-GAGGATCCCCGGGTACCGG-TCTGGAGGCTTGCTGAAGGC-General Biol Company; miR-330-5p inhibitor-CACACATTCCACAGGCTAGC-GGGCCATTTGTTCCATGTG-General Biol Company; hFBXO21 si-1-AUGUAGUCAAAGAUGUCCAGGTT-CCUGGACAUCUUUGACUACAUTT-General Biol Company; hFBXO21 si-2-GCAGGUGAAUCAUCCAUGAUATT-UAUCAUGGAUGAUUCACCUGCTT-General Biol Company.

### RNA extraction, reverse transcription and quantitative real-time PCR (qRT-PCR)

Total RNA was extracted from fresh tissues or ccRCC cell lines using Trizol reagent (Thermo Fisher Scientific, USA). The extracted RNA was reverse transcribed into cDNA using either the Evo M-MLV RT Mix Kit (Accurate Biology, China) or the miRNA 1 st strand cDNA synthesis kit (Accurate Biology, China). Next, we carried out qRT-PCR by using SYBR® Green Premix Pro Taq HS qPCR Kit with Rox Reference Dye (Accurate Biology, China). Three multiple holes were set for each experiment and each measurement was performed in triplicate. The housekeeping gene GAPDH as the reference and relative expression levels of the target genes were calculated using the 2-ΔΔCT calculation method. All the primer sequences used in this research were listed in Supplemental Table 2.

### Nuclear cytosol fractionation experiment

Cell pellets were collected, and cytoplasmic and nuclear RNA were extracted separately using a Nuclear Cytosol Fractionation Kit (Norgen, Canada). Reverse transcription and qPCR were performed on the extracted RNA, and the relative expression levels of the target gene in the nucleus and cytoplasm were calculated separately according to the protocol mentioned in the previous section.

### Fluorescence in situ hybridization (FISH)

The FISH was performed to detect the sub-localization of circGRHL2 in ccRCC cells. Sterilized 24 × 24 mm cover glasses in a 6-well plate, and ccRCC cells were seeded at a density of 10^4^ cells per well. On the following day, cells were fixed with 4% paraformaldehyde (Servicebio, China) for 20 min, followed by permeabilization with 0.1% TritonX-100 (Solarbio, China) for 15 min. The circGRHL2 probe was diluted with hybridization buffer and incubated with cells at 37℃ overnight. After hybridization, the cover glasses were removed, and 3% BSA (Sparkjade, China) solution was added to the cells and incubated for 60 min. Nuclei were stained with DAPI (Solarbio, China), and images were captured using a fluorescence microscope (ECHO, USA). For cell co-located FISH assay, Cy3-labeled circGRHL2 FISH probe and CY5-labeled miR-330-5p probe were designed and synthesized by Guangzhou Geneseed Biotech. FISH was conducted using FISH Kit (Geneseed, Guangzhou, China). ACHN cells were fixed with 4% (w/v) paraformaldehyde (PFA), permeated with 0.5% triton X-100 and hybridized with probe at 37 °C overnight. The hybridization buffer was then gradually eluted with 4 × saline-sodium citrate (SSC), 2 × SSC and 1 × SSC. Images were captured using a fluorescence microscope (ECHO, USA).

### Cell counting kit-8 (CCK-8) assay, EdU staining assay and colony formation assay

To evaluate the proliferation capacity of cells in vitro*,* several assays were conducted. For CCK8 assay, cells in logarithmic growth phase were seeded into a 96-well plate at a density of 2 × 10^3^ cells per well. At 24 h, 48 h, 72 h, and 96 h, 10μL of CCK-8 reagent (CG801; Cellgene, China) was added to each well, followed by incubation at 37℃ for 1 h. The absorbance was measured at 450 nm using a spectrophotometer (Thermo Fisher Scientific, USA). For EdU assay, the YF®594 Click-iT EdU Universal Cell Proliferation Detection Kit (C6045M; UElandy, China) was used according to the manufacturer’s instructions. For the colony formation assay, transfected cells in logarithmic growth phase were seeded into a 6-well plate at a density of 1000 cells per well and cultivated for 14 days. The colonies were then fixed in 4% paraformaldehyde for 30 min and stained with Giemsa staining (Solarbio, China) for 20 min. Colonies were counted and photographed at the end of the experiment.

### Wound healing assay

Transfected cells were seeded in 6-well plates at the ratio of 10^6^ per well one day prior to the assay. A horizontally and vertically straight scratch was made on the adherent cells using a 1000 ml sterile pipette tip. After washing cell scum with phosphate buffered saline (PBS), images were immediately captured (0 h point) under the fluorescence microscope. Complete medium was then added to each well, and images were taken again at the same location after 12 h or 24 h. The migration rate was calculated by dividing the migration distance by the original distance, and the cell migration ability was evaluated based on the ratio.

### Matrigel transwell invasion assay

A total of 70 µL diluted matrix gel was placed into the upper chamber of 24-well Transwell, and incubate at 37 °C for 1 h to allow gelation. Cells were resuspended in serum-free medium, and 200 µL supernatant was placed on the Matrigel matrix gel at a density of 5 × 10^4^ cells per well. A total of 600 µL of complete medium was added to the outside of the chamber, and then was incubated at 37 °C for 48 to 72 h. Transwell was removed from the 24-well plate, and cells were fixed with methanol and stained with 0.5% Giemsa staining (Solarbio, China) for 30 min. Non-invasive cells were scraped off at the top of Transwell with a cotton swab and invading cells were counted under an optical microscope.

### Determination of IC50 by sunitininb

Based on changes in cell viability after treatment with Sunitinib, IC50 values were determined using non-linear regression curves fitted to Sunitinib concentration and OD450nm values. The analysis was performed using the Hill Eq [15], where Min and Max represent maximum and mini-mum values of OD450 nm in the sigmoidal regression, respectively. The measured IC50 was calculated using the least square fit of four parameter-sigmoidal curves using Prism version 8 (GraphPad, San Diego, CA, USA).

### Tumorigenesis in vivo and therapy assay

For this study, 6-week-old nude male mice were recruited for the xenograft models. All male mice weighed 20–22 g and were randomly assigned to the experimental group and the control group. Transfected cells, both circGRHL2 overexpression and control were resuspended in 100 µL PBS at a density of 10^8^ cells per milliliter, and then were subcutaneously injected into the armpit of nude mice (*n* = 10). The mass and volume of tumors in nude mice were regularly measured after injection. At the fifth week post-injection, the mice were sacrificed and the tumors were removed for weight measure and photograph record. For therapy assay, the experiment and control group (*n* = 5) receive treatment of sunitinib and vehicle (40 mg/kg), respectively, through oral gavage.

### Dual‑luciferase reporter assay

Reporter vectors containing the wild-type (WT) and the mutant (MUT) sequence of circGRHL2 and FBXO21 were synthesized by Obio Technology (Shanghai) Corp., Ltd. The constructed luciferase reporter vectors and miR-330-5p mimic were co-transfected into HEK293T cells using Lipofectamine 3000 (Thermo Fisher Scientific, USA). Luciferase activity was determined using a Dual-Luciferase Reporter Assay System Kit (E1960, Promega Corporation, China) after 48 h of transfection.

### Western blot

Cells were lysed using RIPA lysis buffer (Servicebio, China) with a protease inhibitor cocktail (MedChemExpress, USA), and the supernatant protein was obtained by centrifugation. Protein concentration was determined using a BCA protein assay kit (Servicebio, China). Equal amounts of protein extract were loaded onto an SDS-PAGE gel (Vazyme Biotech, China) for separation, and then electronically transferred onto a PVDF membrane (Thermo Fisher Scientific, USA). The membrane was incubated in 5% fat-free milk in PBS for 2 h to block non-specific binding, and was then incubated with a specific primary antibody at 4℃ overnight. On the following day, the membrane was incubated with the corresponding secondary antibody for 1 h at room temperature. The protein bands were visualized with a chemiluminescence imaging analysis system (Clinx, China) and analyzed with ImageJ software.

For endogenous interaction, protein extracts were incubated with FBXO21/P85α antibody or rabbit IgG control (Cell Signaling Technologies, USA) overnight at 4 °C, followed by incubation with Protein A agarose beads (Cell Signaling, #9863) for 3 h at 4 °C. Lysates were incubated with Anti-FBXO21 or Anti-p85α beads as described. Beads were washed, and eluted by boiling in 1 × laemmli buffer for 5 min. The antibodies used were as follows: anti-FBXO21 (rabbit; Abclonal, China; 1:1000), anti-β-ACTIN (rabbit; Abmart, China; 1:1000), anti-E-cadherin (mouse; Cell Signaling Technology, USA; 1:1000), anti-N-cadherin (mouse; Cell Signaling Technology, USA; 1:1000), anti-vimentin (rabbit; Cell Signaling Technology, USA; 1:1000), anti-AKT (rabbit; Cell Signaling Technology, USA; 1:1000), anti-p-AKT (rabbit; Cell Signaling Technology, USA; 1:1000), PI3 Kinase p110α (rabbit; Cell Signaling Technology, USA; 1:1000), PI3 Kinase p85 (rabbit; Cell Signaling Technology, USA; 1:1000), goat anti-rabbit secondary antibody (Cell Signaling Technology, USA, 1:5000), and goat anti-mouse secondary antibody (Cell Signaling Technology, USA, 1:5000), goat anti-mouse ubiquitin Polyclonal antibody (Proteintech, China,1:5000).

### RNA pulldown assay

For RNA pulldown assay, biotinylated-circGRHL2 probe was synthesized by Geneseed (Guangzhou, China), and the oligo probe was used as a control. The Dynabeads™ (Thermo Fisher Scientific, USA) was recruited to pulldown the target biotin-coupled RNA complex according to the manufacturer’s instructions. The RNA complex in beads was extracted by Trizol reagent and analyzed by qRT-PCR.

### Statistical analysis

All the experiment mentioned above were repeated in triplicate, and the data were shown as mean ± standard deviation (Mean ± SD). The Student t-test was used to compare the difference between the two groups, while the ANOVA analysis was used to compare the difference among multiple groups. Chi-squared test was used to analyze the relationship between the gene expression and clinical pathological characters. *P* < 0.05 was considered statistically significant, and all the data analysis and plotting were performed using GraphPad Prism 8 (GraphPad Software, Inc., La Jolla, CA, USA).

## Result

### circGRHL2 is underexpressed in ccRCC and correlates with patient clinicopathological features

**Fig. 1:**
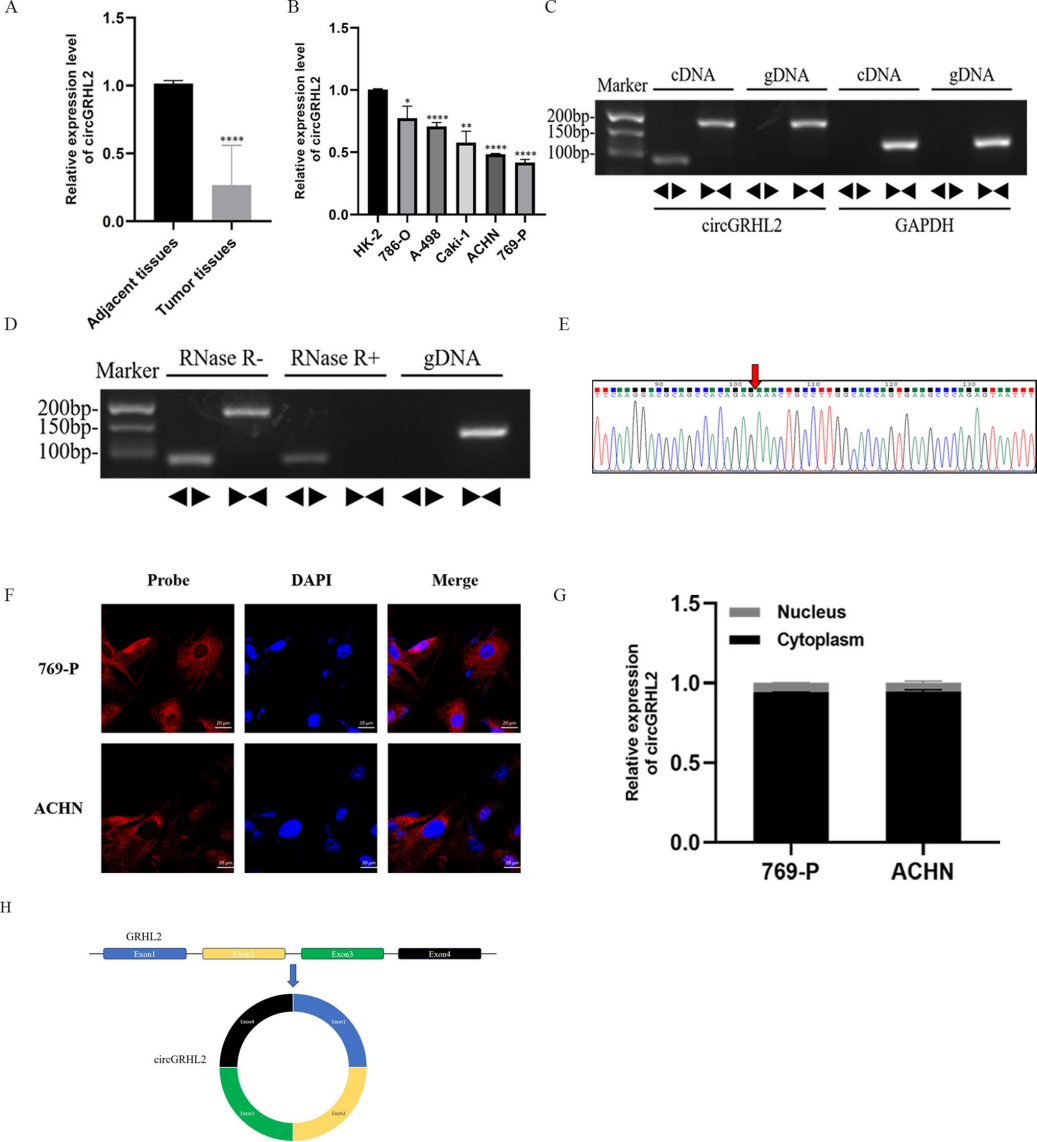
Identification of circGRHL2 in ccRCC tissue and cell lines

Previous work in our group found that circGRHL2 expression in bladder cancer was significantly elevated and plays an important role in tumor progression. Therefore, we were interested in the role of circGRH2 in ccRCC. To explore the expression of circGRHL2 in ccRCC and its relationship with the clinicopathological characteristics of patients, we collected tumor tissues and corresponding adjacent normal tissues of ccRCC patients (*n* = 100), and quantified circGRHL2 expression by qRT-PCR. The results showed that circGRHL2 was significantly underexpressed in ccRCC tissues (Fig. 1A). At the same time, we analyzed the expression of circGRHL2 in ccRCC cell lines 786-O, 769-P, ACHN, A498, Caki-1 and human renal tubular epithelial cell HK-2. The expression of circGRHL2 was lower in ccRCC cell lines compared with HK-2 cells (Fig. 1B). Subsequently, we divided the patients into high-expression group (*n* = 50) and low-expression group (*n* = 50) according to the median expression of circGRHL2 among all patients. Statistical analysis results showed that the expression of circGRHL2 was significantly correlated with clinical T stage and pathological Fuhrman grade (*p* < 0.001), but not with other clinicopathological characteristics such as age, gender, and presence of lymph node metastasis (Table 1). The circGRHL2 was produced from exons 1 to 4 of the GRHL2 mRNA with a total length of 394 nucleotides. The back-spicing junction point sites of circRNA were determined by Sanger sequencing (Fig. 1E and H). Both convergent and divergent primers were used to verify the circular structure; convergent primers can amplify both genomic DNA and cDNA, while divergent primers selectively amplified cDNA (Fig. 1C). The expression level of GRHL2 mRNA was significantly decreased after RNase R treatment, while the expression level of circGRHL2 exhibited negligible change, indicating that circGRHL2 had a typical circular structure, was resistant to RNase R enzyme degradation (Fig. 1D). Nuclear-cytoplasmic separation assay and FISH tests showed that circGRHL2 was mainly expressed in the cytoplasm (Fig. 1F-G). According to these results, circGRHL2 is a circular RNA mainly located in the cytoplasm, and its lower expression level in RCC tissue suggests that it may has antitumor biological function.

**Table 1:** Correlation between circGRHL2expression and clinicopathologic characteristics

Characteristics	Total	circGRHL2 expression		*p-*value	FDR
	*N* = 100	low(*N* = 50)	high(*N* = 50)		
Age				0.23	0.92
≥ 60	47(47.0%)	27(54.0%)	20(40.0%)		
< 60	53(53.0%)	23(46.0%)	30(60.0%)		
Gender				0.63	1
female	23(23.0%)	10(20.0%)	13(26.0%)		
male	77(77.0%)	40(80.0%)	37(74.0%)		
Pathologic_T				< 0.0001^****^	< 0.0001^****^
T1	71(71.0%)	27(54.0%)	44(88.0%)		
T2	10(10.0%)	5(10.0%)	5(10.0%)		
T3	19(19.0%)	18(36.0%)	1(2.0%)		
Pathologic_N				0.48	1
negative	98(98.0%)	48(96.0%)	50(100.0%)		
positive	2(2.0%)	2(4.0%)	0(0)		
Pathologic_M					
none	100(100.0%)	50(100.0%)	50(100.0%)		
Fuhrman grade				< 0.001^***^	< 0.01^**^
Ⅰ	9(9.4%)	3(6.0%)	6(9.0%)		
Ⅱ	65(67.7%)	26(52.0%)	39(78.0%)		
Ⅲ	20(20.8%)	18(36.0%%)	2(4.0%)		
Ⅳ	2(2.1%)	2(4.0%)	0(0)		

*T*, Tumor grade; *N*, Lymph node; *M*, Metastasis; *FDR*, False discovery rate

Chi-square test,** p < 0.01, ***p <0.001, **** p < 0.0001

### CircGRHL2 inhibits the proliferation, migration and invasion of ccRCC in vitro and in vivo

**Fig. 2:**
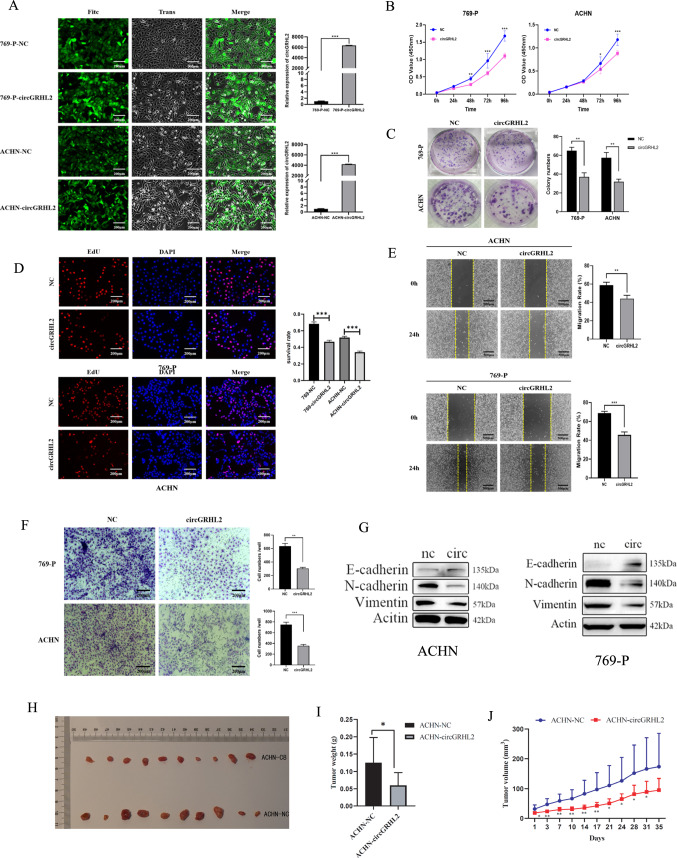
CircGRHL2 inhibits ccRCC cell proliferation, invasion, and migration in vitro and in vivo

In order to explore the biological function of circGRHL2 in ccRCC, we constructed ccRCC cell lines 769-P and ACHN stably overexpressing circGRHL2 by transfecting lentivirus. The transfection efficiency of circGRHL2 was measured by qRT-PCR. The results showed that the expression level of circGRHL2 in overexpressed group was significantly higher than that in blank vector (NC) group (Fig. 2A). The results of CCK-8 experiment showed that the proliferation ability of cells in the overexpression group was significantly reduced (Fig. 2B). Plate colony formation experiments showed that the size and number of cell clusters formed in the circGRHL2 group were smaller and less than NC group (Fig. 2C). EdU experiments showed that the proportion of proliferating cells in the circGRHL2 overexpression group was significantly reduced (Fig. 2D). The above results indicated that overexpression of circGRHL2 could significantly inhibit the proliferation ability of ccRCC cells. Wound healing experiment showed that the migration rate of cells in the circGRHL2 group was significantly weakened (Fig. 2E). Transwell invasion assay showed that the number of cells passed through matrigel was significantly reduced in the circGRHL2 overexpression group (Fig. 2F). At the same time, after overexpression of circGRHL2, the EMT of ACHN and 769-P cells was significantly inhibited (Fig. 2G). Next, we explore the effect of circGRHL2 on the proliferation ability in vivo, we inoculated the above-mentioned ACHN cells stably overexpressing circGRHL2 and control cells (ACHN-NC) into the right axilla of nude mice for in vivo tumorigenesis experiments. Thirty-one days after the inoculation, the nude mice were sacrificed and the tumors were dissected and removed (Fig. 2H). The results showed that the tumor volume and mass were significantly lower than those in NC group (Fig. 2I and J). In summary, circGRHL2 significantly inhibit the progression of ccRCC cells in vitro and in vivo.

### Silencing circGRHL2 promotes proliferation, migration and invasion of ccRCC cells

**Fig. 3:**
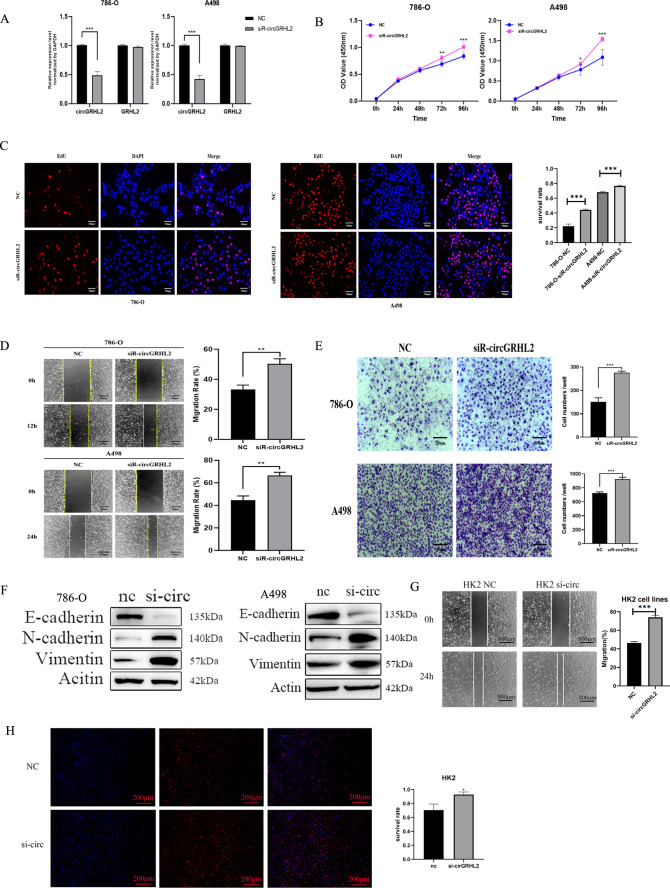
Knock down CircGRHL2 promotes ccRCC cell proliferation, invasion, and migration in vitro

To further investigate the biological function of circGRHL2, we constructed a small interfering RNA (siR-circGRHL2) targeting circGRHL2.The expression of circGRHL2 was quantified by qRT-PCR 48 h after transfection. Silencing circGRHL2 significantly reduce the expression of circGRHL2 in 786-O and A498 cell lines, while the expression level of GRHL2 mRNA remained no change (Fig. 3A). We detected the proliferation ability of cells by CCK-8 assay and EdU assay, and the results showed that the proliferation ability of 786-O and A498 cells was enhanced after silencing circGRHL2 (Fig. 3B and C). Compared with the NC group, the 24 h migration ability of cells after silencing circGRHL2 was significantly increased (Fig. 3D). The invasive ability of 786-O and A498 cells after silencing circGRHL2 was significantly higher than that of NC group (Fig. 3E). Correspondingly, when circGRHL2 was knocked down, the EMT of cells was enhanced (Fig. 3F). We also examined the effect of circGRHL2 on the function of HK2 cells. Knockdown of circGRHL2 also promoted the migration and proliferation abilities of HK2 cells (Fig. 3G and H). The above experimental results indicated that silencing circGRHL2 could significantly promote the proliferation, migration and invasion of ccRCC cells in vitro.

### CircGRHL2 acts as a miR-330-5p sponge in ccRCC cells

**Fig. 4:**
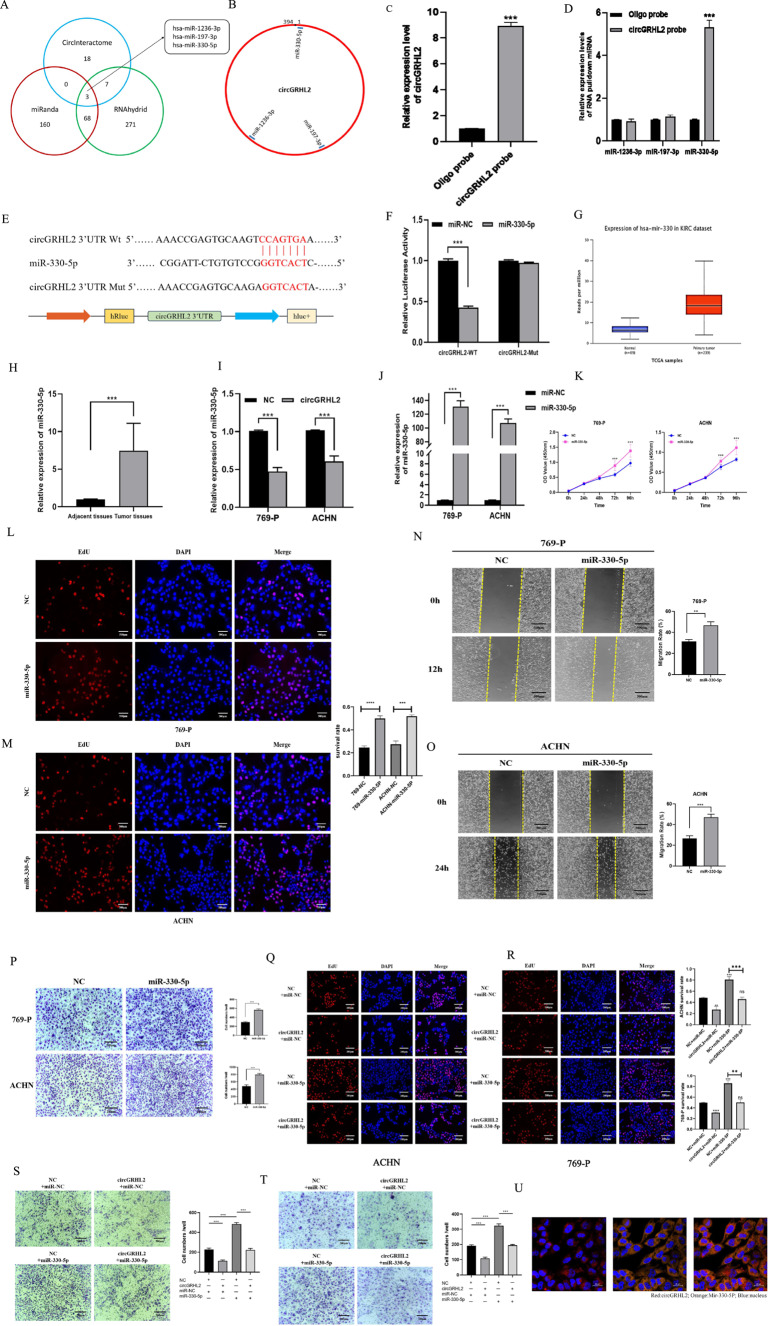
CircGRHL2 serves as a miR-330-5p sponge

CircRNAs located in the cytoplasm mainly exert biological functions by influencing miRNA to regulate gene expression [16]. Therefore, we hypothesised that circGRHL2 may play a role in tumor suppression by acting as a miRNA sponge. The potential downstream miRNAs of circGRHL2 were predicted according to miRanda, RNAhydrid, and CircInteractome databases, and miR-1236-3p, miR-197-3p and miR-330-5p were screened out as potential targets bind to circGRHL2 (Fig. 4A and B). We constructed biotin-labeled circGRHL2 probes and Oligo probes for RNA pulldown experiments, and detected the relative expression levels of three potential target miRNAs in the pulled-down RNA complexes by qRT-PCR. The results showed that the expression level of miR-330-5p was significantly increased compared with the Oligo probe, suggesting that miR-330-5p might bind to circGRHL2 (Fig. 4C and D). We performed a dual-luciferase reporter assay to further verify whether circGRHL2 binds to miR-330-5p directly. The results showed that compared with the control group, miR-330-5p significantly reduce the relative luciferase activity of circGRHL2-WT cells but had no inhibitory effect in circGRHL2-mutation cells (Fig. 4E and F). FISH experiments also demonstrated that circGRHL2 could directly bind to miR-330-5p (Fig. 4U). The results of the TCGA database showed that miR-330-5p was significantly highly-expressed in ccRCC compared with adjacent normal tissues (Fig. 4G). We used 20 pairs of ccRCC tissues and adjacent normal tissues for verification, and comparable results were obtained (Fig. 4H). Furthermore, we found that the expression of miR-330-5p was also decreased in 769-P and ACHN cells after overexpressing circGRHL2 (Fig. 4I). Then we transfected the blank vector (miR-NC) and miR-330-5p into 769-P and ACHN cell lines and performed cell functional experiments. Quantitative RT-PCR results showed that the expression of miR-330-5p was significantly increased after transfection (Fig. 6A). Overexpression of miR-330-5p significantly promote the proliferation (Fig. 4K and M), migration (Fig. 4N and O) and invasion abilities (Fig. 4P) of ccRCC cells. To clarify the effect of miR-330-5p on the tumor suppressor regulated by circGRHL2, we simultaneously transfected miR-330-5p and circGRHL2 into ACHN and 769-P cell lines. Compared with transfection of circGRHL2 alone, miR-330-5p reversed its ability to inhibit the proliferation (Fig. 4Q and R) and invasion (Fig. 4S and T). The above experimental results indicated that miR-330-5p promotes RCC cells growth and reverse the tumor suppression effect caused by circGRHL2.

### FBXO21 is a direct target protein of miR-330-5p

**Fig. 5:**
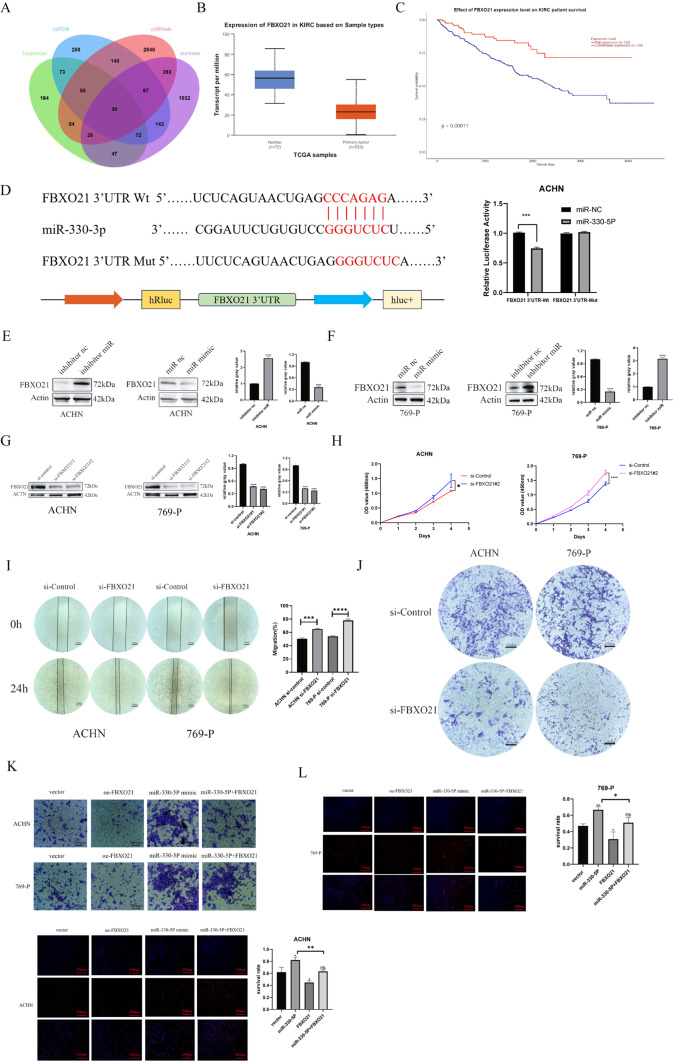
FBXO21 is a direct target protein of miR-330-5p

To identify the downstream target genes of miR-330-5p, we searched four online databases for prediction: starbase3.0, TargetScan, MirWalk, and miRDB. Thirty genes (supplement Table 2) in the intersection of the four databases could be negatively regulated by miR330-5p (Fig. 5A). Through database verification, we found that 4 of the 30 molecules had low expression in renal cancer. TCGA database showed that FBXO21 was expressed at low level in renal cancer and was associated with poor prognosis (Fig. 5B and C). To determine whether miR-330-5p directly bind to the 3’UTR of FBXO21, a dual-luciferase reporter experiment was conducted. The miR-330-5p mimic directly inhibited the luciferase activity of cells containing WT FBXO21 3’UTR, while no change was observed in mutant cells (Fig. 5D). Western blot results also showed the opposite expression change of FBXO21 after transfecting miR-mimic and inhibitor (Fig. 5E and F). After FBXO21 knockdown, the proliferation, migration and invasion abilities of ccRCC cells were enhanced (Fig. 5G-J). After overexpression of miR-330-5p, the invasion and migration ability of 769-P were enhanced. Meanwhile, overexpression of FBXO21 could rescue the promoting effects caused by miRNA (Fig. 5K and L). The above results indicated that FBXO21 was a direct target protein of miR-330-5p and can reverse the action of miR-330-5p.

### CircGRHL2/miR-330-5p/FBXO21 inhibits EMT via PI3K/AKT pathways

**Fig. 6:**
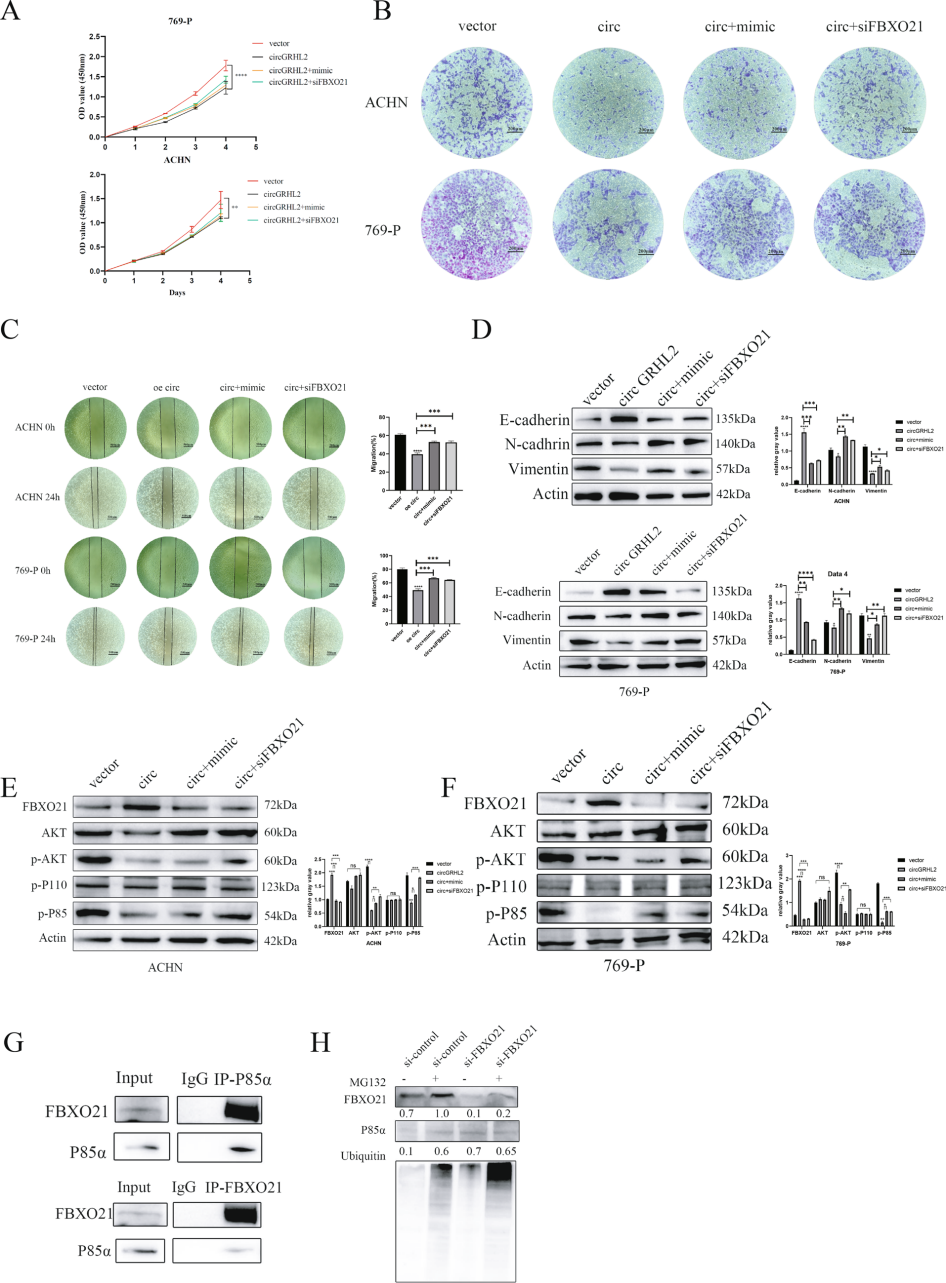
CircGRHL2/miR-330-5p/FBXO21 inhibits EMT via PI3K/AKT pathways.

To further elucidate the function of the circGRHL2/miR-330-5p/FBXO21 axis, we simultaneously transfected miR-330-5p mimics or siR-FBXO21 in cells overexpressing circGRHL2. The results showed that overexpression of miR330-5p and knockdown of FBXO21 reversed the circGRHL2-overexpression induced inhibitory effects on of, including proliferation and invasion (Fig. 6A-C). FBXO21 is a ubiquitin E3 ligase that negatively regulate the PI3K/AKT pathway by ubiquitinating and degrading the regulatory subunit P85α of PI3K [17]. Studies have shown that FBXO21 inhibited autophagy and EMT to act as a tumor suppressor [18, 19]. We found that after up- or down-expression of circGRHL2, renal cancer EMT markers were changed in opposite trend (Figs. 2G and 3F). On the contrary, autophagy markers were not affected by circGRHL2 (Fig. S1). Given that circGRHL2 regulated the expression of FBXO21 through miRNA, we speculated that circGRHL2 affected EMT through FBXO21. We found that after transfection of miR-330-5p in ACHN cells overexpressing circGRHL2, the changes in EMT were reversed. Similarly, knocking down FBXO21 could reverse the effects of overexpression circGRHL2 on EMT in ACHN cells (Fig. 6D). The same results were also found in 769 cells (Fig. 6D). Next, we transfected miR-330-5p mimic and siR-FBXO21 in ACHN cells overexpressing circGRHL2. MiR-330-5p mimic and siR-FBXO21 attenuate the expression of FBXO21 (Fig. 6E and F). The changes in FBXO21 did not affect the total amount of AKT and phosphorylated P110, but were positively correlated with the activation of P85. CircGRHL2 inhibited the phosphorylation of P85 and the activation of the AKT signaling pathway. MiR-330-5p upregulated the AKT pathway, while FBXO21 had the opposite function (Fig. 6E and F). Next, we confirmed through IP experiments that FBXO21 can bind to P85 (Fig. 6G). In the control group, after adding MG132 to inhibit the proteasome, the ubiquitination level and the expression of P85 increased. After knocking down FBXO21, MG132 did not significantly change the expression level of P85α, indicating that the effect of FBXO21 on P85exerted through ubiquitination. Overall, knocking down FBXO21 can increase the ubiquitination of 769-P cells (Fig. 6H). The above results indicated that circGRHL2 regulated the expression of FBXO21 by sponging miR-330-5p, thereby affecting the activation of the PI3K/AKT signaling pathway and inhibits EMT of ccRCC.

### CircGRHL2 inhibits tumor growth and promote the anti-tumor effect of sunitinib in vivo

**Fig. 7:**
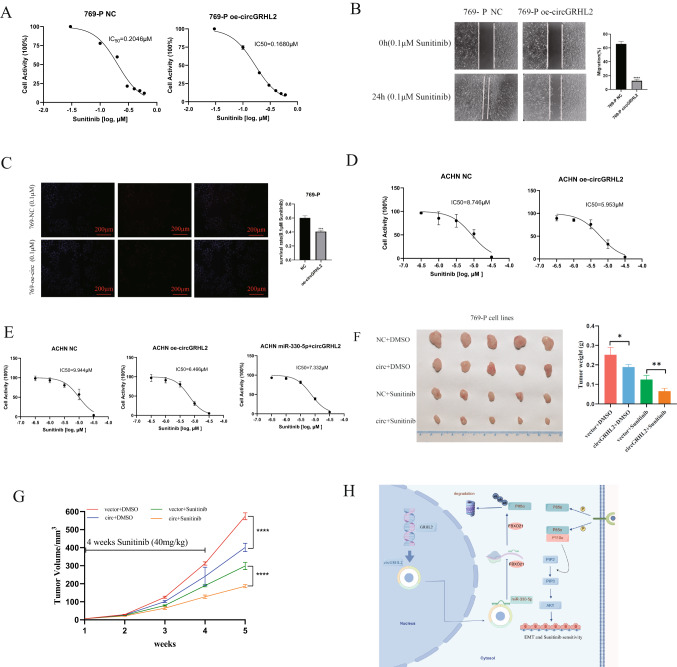
CircGRHL2 inhibits tumor growth and promote the anti-tumor effect of sunitinib in vivo.

In previous studies, we found that circGRHL2 inhibited the tumorigenicity of mouse subcutaneous ACHN cells, and its overexpression suppressed EMT. Since EMT promotes the malignant phenotype and affects the uptake of drugs by tumor cells, it might also affect the response to antitumor drugs [20]. Therefore, we aimed to determine whether circGRHL2 could enhance the sensitivity of chemotherapeutic drugs. Sunitinib, a tyrosine kinase inhibitor, was selected as the representative drug for this investigation. Studies have shown that in sunitinib-resistant RCC cells, the AKT signaling pathway is continuously activated and the EMT transformation is more severe [21]. Therefore, we overexpressed circGRHL2 in 769-P cells and performed sunitinib drug sensitivity test. The results showed that after overexpression of circGRHL2, the IC50 of 769-P cells decreased by 18.89% compared with WT cells (0.1680 μM vs 0.2046 μM) (Fig. 7A). In 0.1 μM sunitinib, circGRHL2 could inhibit the proliferation and migration of 769-P cells (Fig. 7B and C). We performed the same experiment on ACHN cells, and overexpression of circGRHL2 reduced the IC50 value of ACHN cells. Furthermore, we also found that overexpression of miR-330-5p could rescue the reduction in IC50 caused by circGRHL2 (Fig. 7D and E). In addition, the tumorigenicity of 769-P cells decreased in vivo after overexpression of circGRHL2 (Fig. 7F), and compared with the control group, the therapeutic sensitivity of sunitinib was significantly enhanced after overexpression circGRHL2. Sunitinib and circGRHL2 combined treatment obtained the lowest tumor weight (g) and volume among four subgroups (Fig. 7F and G). The above results indicated that circGRHL2 inhibited EMT of ccRCC and increased the sensitivity of tumor cells to sunitinib.

## Discussion

The development of high-throughput sequencing technology shows that circRNA is widely expressed in different tissues of the human body and is related to the occurrence and development of various tumors [16]. The circRNA has a circular structure and is naturally more stable, which is convenient for detection and evaluation of the relationship between expression and disease. The transcription factor Grainyhead-like 2 (GRHL2) is a key transcription factor of epithelial tissue, playing different roles in promoting and suppressing cancer in different cancers. The expression of GRHL2 is positively correlated with the expression of the key EMT factor E-cadherin [22]. In breast cancer, GRHL2 regulates the balance and growth of luminal and basal tumors through EMT. [23]. Until now, there is blank in the knowledge of the role of circGRHL2 in tumors. Our study revealed that circGRHL2 has low expression in ccRCC and proved that it could inhibit tumor proliferation, migration and invasion in ccRCC. At the same time, we found that after its highly expression, ccRCC cells expressed more epithelial phenotypes and could inhibit EMT.

Given that circGRHL2 is expressed in the cytoplasm and lacks a ribosome entry site or N6 promoter encoding a polypeptide, we hypothesized that it may play roles through sponging miRNA. We found that miR-330-5p was directly sponged by circGRHL2. Studies have reported that miR-330-5p, as a "target" of Circ_0000235, down-regulates and activates monocarboxylate transporter 4 (MCT4), promoting glycolysis and the growth of bladder cancer [24]. MiR-330-5p can also regulate the progression of pancreatic cancer and ovarian cancer by targeting the expression of PAK1 (p21-activated kinase 1) and cytoplasmic polyadenylation element-binding protein 4 (CPEB4) [25, 26]. In our study, miR-330-5p has the positive effect of promoting ccRCC proliferation, it is capable of reversing the effect of circGRHL2. MiRNA inhibits gene expression by targeting the 3′UTR of post-transcriptional mRNA. Through multiple database screening and Western Blot verification, we identified the target of miR-330-5p, F-box ubiquitin E3 ligase, FBXO21. FBXO21 is one of the subunits of ubiquitin protein ligase, which plays a role in phosphorylation-dependent ubiquitination. FBXO21 negatively regulates the PI3K/AKT/ERK pathway by degrading the subunit P85α of PI3K, affecting the progression of leukemia [17]. FBXO21 also mediate the polyubiquitination and degradation of KRT16, affecting EMT and inhibiting the progression of lung cancer [27]. Co-immunoprecipitation shows FBXO21 can directly bind to P85α, degrading P85α through ubiquitination, and inhibit the activation of the PI3K signaling pathway.

Sunitinib, a first-line drug for ccRCC, showed obvious protein kinase B (AKT) inhibition and EMT during medication, leading to sunitinib resistance. Thus, in order to develop more effective and long-lasting treatment strategies for advanced ccRCC patients, it will be crucial to ascertain how to overcome sunitinib resistance. Given that both circGRHL2 and FBXO21 can play a role in EMT, and FBXO21 is proved negatively regulate the activation of the PI3K/AKT pathway. CircGRHL2 enhances the drug sensitivity of ccRCC to sunitinib via PI3K/AKT and EMT. The mechanisms that have been shown to produce resistance to sunitinib include: facilitating tumor proliferation and metastasis by increasing the epithelial-to-mesenchymal transition and presence of specific microRNA (miRNA), long non-coding RNA (lncRNA), circular RNA (circRNA) and long intergenic noncoding RNA (lincRNA) [28]. The research on the influence of circRNA regulating EMT on tumor progression is increasing. Chen's research indicated that circNEIL3 as a TGFβ-repressive circRNA, exerted its metastasis-repressive function through its direct interaction with YBX1, which consequently promoted the Nedd4L-mediated proteasomal degradation of YBX1 [29]; circITGB6 enhanced the mRNA stability of PDPN, an EMT-promoting gene by directly interacting with IGF2BP3 and circITGB6 wass found to potently promote EMT process and tumor metastasis in vitro and in vivo [30]. Regulating EMT through circRNA to improve drug sensitivity has significant clinical exploring significance.

In our research, there are some limitations. We have identified clinical tumor samples had the low expression of circGRHL2. However, due to lacking of online databases describing circRNAs and its prognosis significance, and the patients in our cohort are still under follow-up, the relationship between circGRHL2 expression and survival has not been clarified. In addition, when searching for downstream molecules of circGRHL2, high-throughput sequencing was not conducted. Fortunately, we identified the downstream targets through the database.

In summary, circGRHL2 regulates the expression of FBXO21 by sponging miR-330-5p, and then inhibits the activation of the PI3K/AKT pathway by regulating P85 phosphorylation, reduces EMT, and increases the sensitivity of ccRCC to sunitinib. This study not only enriches our understanding of the regulatory mechanism of circGRHL2, but also provides a potential new target for overcoming sunitinib resistance in ccRCC.

## Supplementary Information

Below is the link to the electronic supplementary material.

Supplementary file1 (PDF 44 KB)

Supplementary file2 (XLSX 13 KB)

Supplementary file3 (XLSX 9 KB)

Supplementary file4 (DOCX 15 KB)

## Data Availability

All of the relevant data and materials are freely available to any investigator upon request.
