# Supplementary figures and images for: CircGRHL2 suppresses EMT and enhances sunitinib sensitivity in ccRCC via the miR-330-5p/FBXO21 axis
Source: Cell Mol Life Sci. 2026 Apr 29;83(1):256. doi: 10.1007/s00018-026-06102-7 (PMC13272732; doi:10.1007/s00018-026-06102-7)

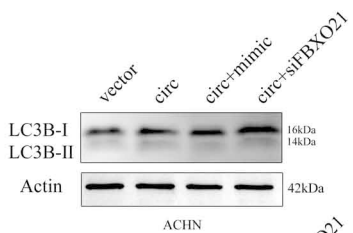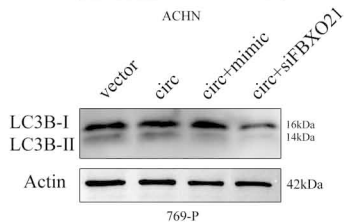

Supplement: Supplementary file 1 — Supplementary file1 (PDF 44 KB) [file 18_2026_6102_MOESM1_ESM.pdf]
