# Supplementary material for: CircGRHL2 suppresses EMT and enhances sunitinib sensitivity in ccRCC via the miR-330-5p/FBXO21 axis
Source: Cell Mol Life Sci. 2026 Apr 29;83(1):256. doi: 10.1007/s00018-026-06102-7 (PMC13272732; doi:10.1007/s00018-026-06102-7)
Supplement: Supplementary file 4 — Supplementary file4 (DOCX 15 KB) [file 18_2026_6102_MOESM4_ESM.docx]

**Supplement Table 3 qRT-PCR** **Primer Sequence**

| **Primer** | **Primer Sequence (5’-3’)** |
| --- | --- |
| circGRHL2-F | GGACAGCACATACAGCGAGAG |
| circGRHL2-R | CCTCCACTCAAATTACTCTGG |
| GAPDH-F | UUCUCCGAACGUGUCACGUTT |
| GAPDH-R | ACGUGACACGUUCGGAGAATT |
| U6 | GGAACGATACAGAGAAGATTAGC |
| hsa-miR-197-3p | CCACCTTCTCCACCCAGCAA |
| hsa-miR-330-5p | UUCUCCGAACGUGUCACGUTT |
| hsa-miR-1236-3p | AACAAGCCTCTTCCCCTTGT |
